# Study on the Physical and Rheological Characterisation of Low-Density Polyethylene (LDPE)/Recycled Crumb Rubber (RCR) on Asphalt Binders

**DOI:** 10.3390/molecules29030716

**Published:** 2024-02-04

**Authors:** Shibo Zhang, Yong Yan, Yang Yang, Rongxin Guo

**Affiliations:** 1 Faculty of Civil Engineering and Mechanics, Kunming University of Science and Technology, Kunming 650500, China; 20212210036@stu.kust.edu.cn (S.Z.); yangyang0416@kust.edu.cn (Y.Y.); guorx@kmust.edu.cn (R.G.); 2 Yunnan Key Laboratory of Disaster Reduction in Civil Engineering, Kunming 650500, China; 3 International Joint Laboratory for Green Construction and Intelligent Maintenance of Yunnan Province, Kunming 650500, China

**Keywords:** polymer processing, waste management, sustainability, crumb-rubber-modified asphalt, rheological properties

## Abstract

Recycled crumb rubber (RCR) is considered a reliable asphalt modifier and a solution to the problem of scrap tyre recycling. RCR-modified asphalt (RCRMA) typically has good low-temperature performance and storage stability. However, the pre-treatment of crumb rubber (CR) impairs its physical properties, resulting in poor high-temperature performance, which limits the industrial application of RCRMA. In this study, low-density polyethylene (LDPE) composite RCR was used to modify asphalt, and LDPE/RCR-composite-modified asphalt (L-RCRMA) was produced to compensate for the deficiencies in the high-temperature performance of RCRMA. The comprehensive physical properties of L-RCRMA were elucidated using tests such as the conventional properties, rotational viscosity, and rheological tests. The results showed that the incorporation of LDPE improved the high-temperature stability and rutting resistance of the asphalt, but an excessive amount of LDPE impaired the low-temperature performance and storage stability of L-RCRMA. Therefore, it is necessary to control the amount of LDPE to balance the performance of the asphalt. On this basis, we recommend a dosage of 20% for RCR and 1.5% for LDPE.

## 1. Introduction

With the increasing traffic volumes worldwide and the inherent performance deficiencies of asphalt pavements, the service lives of these pavements are reduced [1,2]. At the same time, the increase in used tyres has added pressure on environmental protection and waste recycling [3]. LDPE is a plastic material with good processability, but its chemical stability makes LDPE waste difficult to degrade naturally and to recycle [4]. Many studies at home and abroad have proven the feasibility of polymer-modified asphalt, so the application of CR composite LDPE for asphalt modification has become a new trend in sustainable development [5,6,7,8].

Relevant studies have confirmed that the addition of CR can significantly improve asphalt’s high-temperature performance and resistance to permanent deformation, but it will adversely affect the low-temperature performance of asphalt, and there are density differences and thermodynamic incompatibilities between CR and matrix asphalt, resulting in the poor storage stability of modified asphalt [9,10,11]. Due to its softness and elongation, LDPE tends to react with asphalt during processing to form new cross-linked structures, thus improving the high-temperature stability and temperature sensitivity of modified asphalt and its mixes [12]. Studies have shown that the addition of LDPE does not effectively help asphalt’s low-temperature performance, but if the dosage of LDPE exceeds a certain amount, it will play a role in enhancing the toughening of asphalt [13]. However, LDPE also has the problem of poor compatibility with matrix asphalt, which hinders the progress of industrialisation of CR/LDPE-modified asphalt.

Inspired by terminal blend (TB) technology, researchers have attempted to pre-treat CR and LDPE to address the above issues [14,15]. These include, but are not limited to, screw extruders, microwave activation, and microbial depolymerisation techniques [16]. D. Lo Presti et al. [17] added granular rubber particles as asphalt modifiers to release the full potential of liquid polymers by catalytically pre-treating the rubber particles. The results showed that the compatibility between the rubber powder and the asphalt could be significantly improved by maintaining the solubility value of the rubber powder and improving the low-temperature properties of the asphalt based on rubber powder dosages of up to 30%. Yang et al. [18] used Cole–Cole curves to observe the compatibility between CR and asphalt. In general, the compatibility between CR and asphalt is determined by the symmetry of the Cole–Cole plot. A perfectly symmetric semicircular plot indicates better compatibility between the two, while deviations from symmetry indicate incompatibility. The results of the study show that the Cole–Cole curve demonstrates that the pre-treatment of CR has a positive effect on improving compatibility. Yan et al. [19] used ethylene vinyl acetate copolymer (EVA) and LDPE for blending to improve the polarity of LDPE in asphalt, which improved the compatibility between LDPE and asphalt and the storage stability of the modified asphalt. However, the existing studies have a vague knowledge of the preparation process of CR and LDPE pre-treatment, and the rheological properties of pre-treated LDPE/CR-composite-modified asphalt have not been clearly investigated.

In conclusion, in this paper, CR was pre-treated, and composite LDPE was applied for asphalt modification. Based on a conventional asphalt test and a rheological test to characterise the comprehensive physical properties of L-RCRMA, the optimal dosage of pre-treated RCR and LDPE was clarified to provide a reference for the feasibility of industrial application of L-RCRMA.

## 2. Results and Discussion

### 2.1. Routine Performance Characterisation

**Figure 1:**
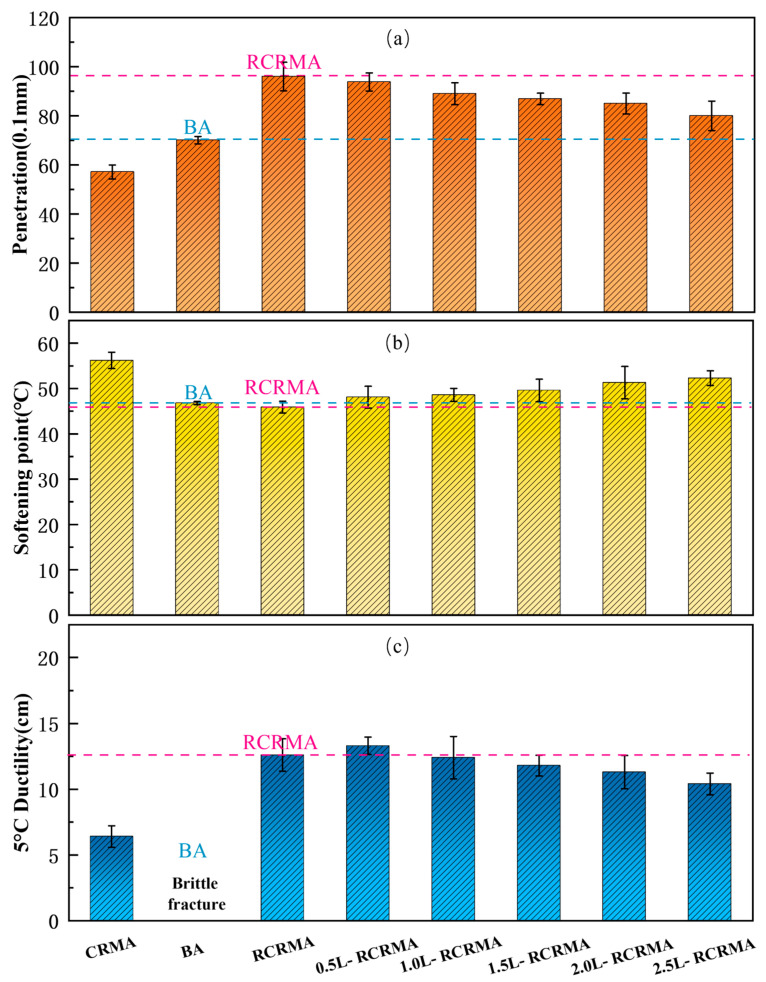
Conventional physical properties of modified asphalt: (**a**) penetration, (**b**) softening point, and (**c**) 5 °C ductility.

As shown in Figure 1a, the penetration value of RCRMA is the largest, indicating that CR damages the hardness of the asphalt after pre-treatment, while the added naphthenic oil softens it [20]. The penetration of L-RCRMA showed a decrease compared with that of RCRMA, and with increasing LDPE doping, the penetration of L-RCRMA decreased. This indicates that the addition of LDPE improves the hardness of the asphalt and compensates for the lack of performance after CR pre-treatment, so that the softness and hardness of the asphalt can be balanced by controlling the dosage of LDPE. The softening point and penetration are related; as shown in Figure 1b, the softening point of RCRMA was the lowest, indicating that the pre-treatment of CR to make the asphalt softer also had some adverse effects on its high-temperature performance and was even lower than that of the matrix asphalt. However, with the addition of LDPE, the softening point of the modified asphalt increased significantly and was already higher than that of the matrix asphalt at a dosage of 0.5%. As the dosage of LDPE increased, the softening point gradually increased, indicating that LDPE can improve the high-temperature performance of asphalt. As shown in Figure 1c, RCRMA had good low-temperature properties, indicating that the addition of naphthenic oil and the pre-treatment of CR can improve the low-temperature properties of asphalt. However, with the increase in LDPE doping, the 5 °C ductility performance of L-RCRMA became progressively worse, indicating that the addition of LDPE is detrimental to the low-temperature performance of asphalt. In line with previous studies, Usman Ghani et al. [21] evaluated the morphology, rheological and dynamic viscosity, and the creep properties of waste HDPE- and LDPE-modified asphalt binders and concluded that the addition of HDPE and LDPE reduces the phase angle of the asphalt, thereby increasing the resistance of the modified asphalt to permanent deformation. Jasim Nisar et al. [22] used LDPE and styrene–butadiene–styrene (SBS) together for the compound modification of asphalt binders, which significantly improved the rutting factor and rotational viscosity of the modified asphalt, indicating that LDPE has a favourable effect on the high-temperature properties of asphalt. From this, it can be concluded that the high- and low-temperature properties of the modified asphalt are balanced by controlling the levels of RCR and LDPE according to the production method described in this paper.

### 2.2. Ease of Construction

Rotational viscosity reflects, to some extent, the ease of construction of asphalt and its binders and is closely related to the difficulty of asphalt construction [23]. In this paper, the change in viscosity of each asphalt sample was investigated over a range of construction temperatures from 135 °C to 175 °C.

**Figure 2:**
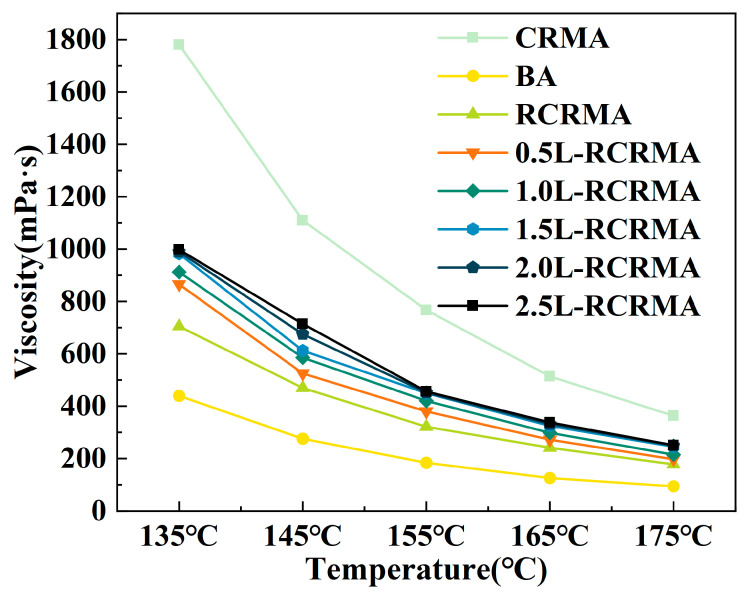
Viscosity–temperature curve.

As shown in Figure 2, CRMA had the highest viscosity due to the larger particle size and heavier mass of untreated CR, which, in the form of small solid particles applied directly to the asphalt modification, greatly increased the density of the asphalt [24]. With the addition of LDPE, the viscosity of L-RCRMA increased, but at higher temperatures above 145 °C, the viscosity of 0.5L-RCRMA and 1.0L-RCRMA was very close to that of RCRMA. The SHRP specification for asphalt binder performance sets the technical requirement that the viscosity of modified asphalt should not exceed 3000 mPa·s at 135 °C. As shown in Figure 2, even the viscosities of CRMA and 2.5L-RCMA, which had the highest viscosities, were still well below 3000 mPa·s at 135 °C, and the viscosities of all samples continued to decrease with increasing temperatures. Therefore, most of the L-RCMAs can still meet the requirements of road construction. From the conventional tests for the penetration, softening point, ductility, and rotational viscosity of asphalt, it can be seen that the maximum penetration difference between the L-RCRMA samples is 13.8 (0.1 mm), the maximum softening point difference is 4.2 °C, and the maximum 5 °C ductility difference is 2.9 cm. Among them, the indicators of 1.0L-RCRMA are close to those of 0.5L-RCRMA, and the indicators of 2.0L-RCRMA are closer to those of 1.5L-RCRMA, with the same trend of change. Therefore, from the perspective of the rational use of resources, we consider 0.5L-RCRMA, 1.5L-RCRMA, and 2.5L-RCRMA to be more representative.

### 2.3. Rheological Properties

#### 2.3.1. Temperature Scanning

**Figure 3:**
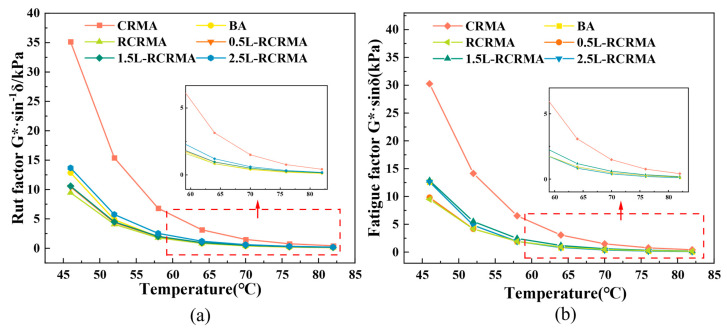
(**a**) Rutting factor. (**b**) Fatigue factor.

As shown in Figure 3a, the incorporation of RCR and LDPE improved the rutting factor of L-RCRMA, suggesting a beneficial effect on the high-temperature stability of the asphalt [25]. The rutting factor of the samples increased with increasing LDPE doping based on RCR addition. Among them, the rutting factors of 0.5L-RCRMA and 1.5L-RCRMA were closer to but better than that of RCRMA. When the dosage reached roughly 2.5%, the rutting factor of the composite-modified asphalt increased significantly and was obviously better than that of the matrix asphalt. When the temperature was lower than 64 °C, the rutting factors of 0.5L-RCRMA and 1.5L-RCRMA were between those of RCRMA and matrix asphalt, and when the temperature was higher than 64 °C, the rutting factor of each dosage of L-RCRMA was higher than that of RCRMA and matrix asphalt, which indicates that when the dosage of LDPE is 0.5–2.5%, it will result in the high-temperature stability of the composite-modified asphalt. At higher temperatures, the improvement effect is more significant.

The fatigue factor is commonly used to assess the susceptibility to fatigue cracking at intermediate asphalt temperatures. In general, the lower the rate of energy loss in the specimen material, i.e., the lower the fatigue factor, the better the fatigue resistance of the specimen. The fatigue factor of the modified asphalt is shown in Figure 3b, and the fatigue factor of CRMA is the largest, indicating that the direct application of waste rubber powder for asphalt modification will cause the modified asphalt to have certain performance defects. The fatigue factor curves of BA, 0.5L-RCRMA, and 2.5L-RCRMA overlap more, indicating that the fatigue resistances of the three are closer. The fatigue factor curves of RCRMA and 1.5L-RCRMA are more overlapped, and the fatigue factor of 1.5L-RCRMA is the least overlapped, which indicates that the desulphurisation and depolymerisation of waste rubber is beneficial to the fatigue resistance of modified asphalt, and at the same time, the fatigue resistance of modified asphalt can be further improved by controlling the dosage of LDPE.

#### 2.3.2. Frequency Scanning

**Figure 4:**
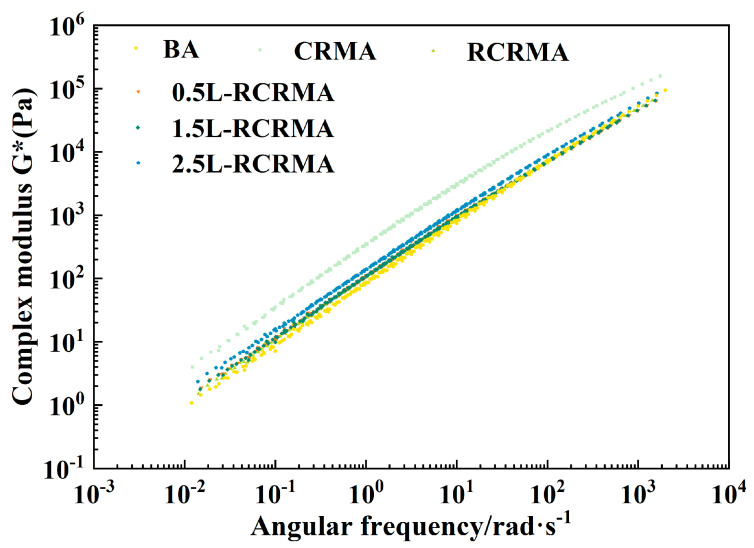
Complex modulus master curve.

As shown in Figure 4, with the incorporation of RCR and LDPE, the complex modulus performance of the composite-modified asphalt showed some changes in both the low-frequency, high-temperature region and the high-frequency, low-temperature region. The complex modulus of L-RCRMA under each LDPE dosage was better than that of the matrix asphalt, and there was a phenomenon of complex modulus increase, indicating that the dosage of LDPE enhanced the ability of the modified asphalt to resist deformation under the action of high temperatures and low speeds and improved the rutting resistance of the modified asphalt to a certain extent.

**Figure 5:**
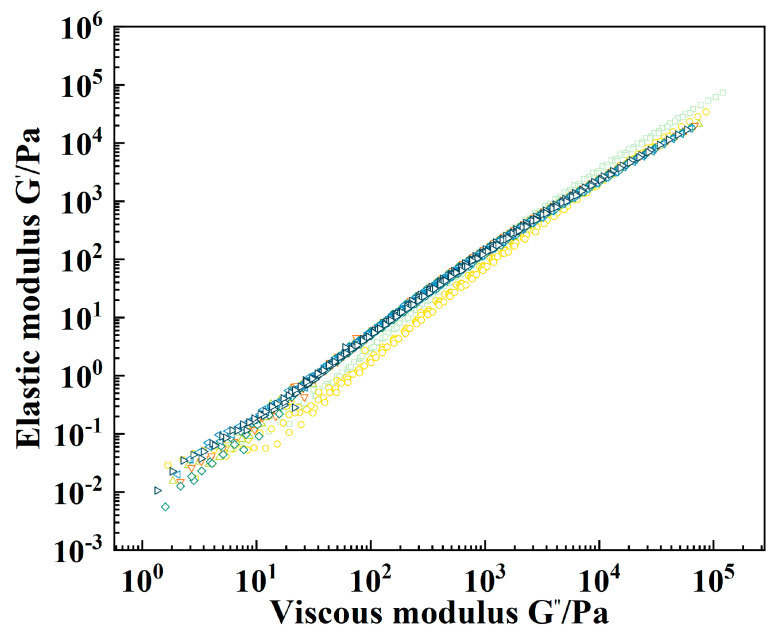

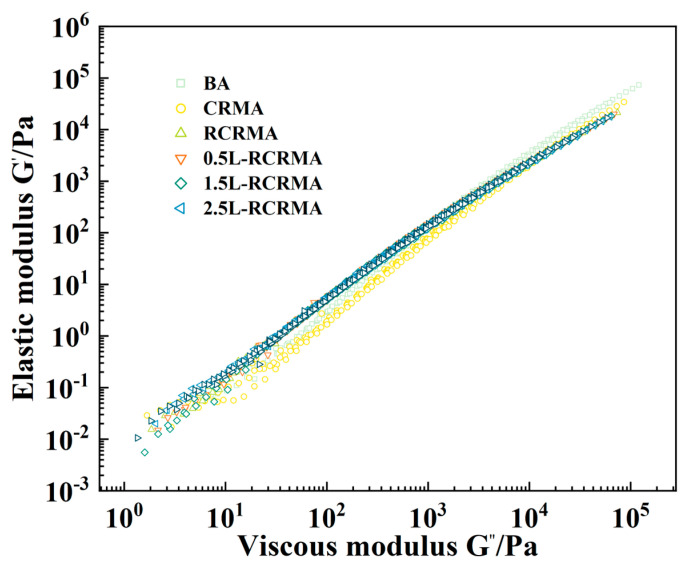
Han curve.

As shown in Figure 5, except for the Han curve distribution of CRMA, the Han curve of L-RCRMA is smooth and continuous, and the slope of the whole curve is excessively smooth and has no plateau area, which indicates that the LDPE doping within 2.5% does not result in any obvious negative effect on the compatibility of RCRMA. This is because RCR and LDPE blended into asphalt at the same time form a continuous network structure with each other, which can make asphalt both more uniformly dispersed and stable and to have better compatibility than either of the modified asphalts alone [26].

#### 2.3.3. MSCR

**Figure 6:**
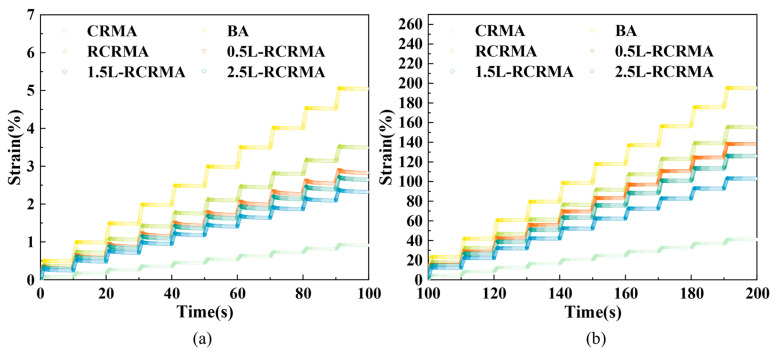
Time–strain curves: (**a**) 0.1 kPa and (**b**) 3.2 kPa.

Figure 6 shows the MSCR results for each asphalt sample. The strains of the modified asphalt at both 0.1 kPa and 3.2 kPa were proportional to time. As shown in Figure 6a, under 0.1 kPa stress, the strain of L-RCRMA was significantly reduced when compared with that of RCRMA and matrix asphalt, indicating that LDPE doping significantly improved the flow deformation resistance of the asphalt. As the LDPE dosage increased, the strain also decreased and showed an obvious regularity. As shown in Figure 6b, the change rule of each asphalt sample under the action of 3.2 kPa stress was basically the same as that under the action of 0.1 kPa, and it is worth noting that the improvement in the asphalt’s ability to resist flow deformation by LDPE was more obvious under the action of 0.1 kPa.

**Figure 7:**
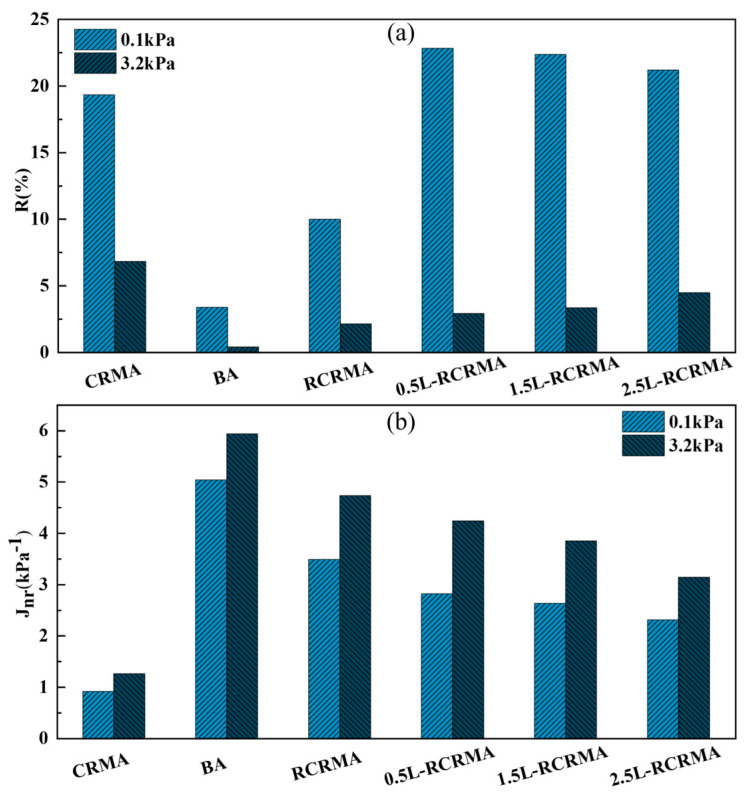
MSCR-based assessment of modified asphalt: (**a**) R and (**b**) *J_nr_*.

The higher the value of the creep recovery rate R, the lower the unrecoverable creep flexibility Jnr, indicating that the elastic deformation capacity of the asphalt is greater. The lower the residual deformation, the better the resistance to high-temperature deformation [27]. As shown in Figure 7a, under 0.1 kPa stress, LDPE doping significantly increased the creep recovery (R) of L-RCRMA, and the creep recovery of each amount of L-RCRMA was higher than that of RCRMA. With the increase in LDPE doping, the creep recovery of each L-RCRMA amount under 0.1 kPa stress slightly decreased, but the difference between them was not obvious. The change rule of each asphalt sample at 3.2 kPa stress was different from that at 0.1 kPa. With the increase in LDPE doping, the creep recovery rate of L-RCRMA at 3.2 kPa showed an increasing trend. Each creep recovery rate of L-RCRMA compared with that of CRMA and matrix asphalt had a very significant advantage but was lower than that of RCRMA. The results show that the incorporation of LDPE can improve the creep recovery rate of asphalt mainly under low stress, and the effect is significant; LDPE can also improve the creep recovery rate under high stress, but this effect is relatively insignificant.

As shown in Figure 7b, the incorporation of LDPE significantly reduced the unrecoverable creep flexibility (*J_nr_*) of L-RCRMA under 0.1 kPa stress, indicating that the incorporation of LDPE significantly improved the ability of the asphalt to resist permanent deformation under repeated loading. As the LDPE loading increased, the unrecoverable creep flexibility of L-RCRMA decreased with apparent regularity. At 3.2 kPa stress, the unrecoverable creep flexibility of each asphalt sample decreased with increasing LDPE loading.

**Figure 8:**
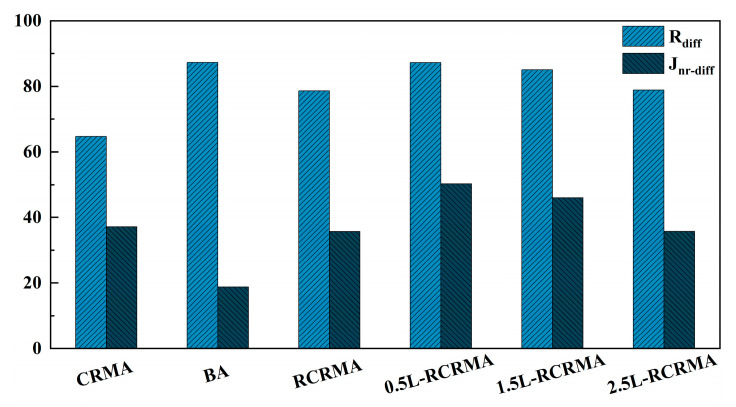
Creep recovery rate difference (R_diff_/%) and unrecoverable creep flexibility difference (J_nr-diff_/%).

As shown in Figure 8, the R_diff_ of L-RCRMA decreased with the increasing LDPE blend. 0.5L-RCRMA performed poorly and similarly to matrix asphalt, but 1.5L-RCRMA and 2.5L-RCRMA performed better, and their creep recovery rate difference was close to that of RCRMA. Meanwhile, LDPE incorporation improved the J_nr-diff_ of L-RCRMA, and the non-recoverable creep modulus difference of L-RCRMA decreased with the increase in LDPE incorporation. This indicates that LDPE incorporation significantly improved the elastic stability of asphalt and reduced the stress sensitivity of asphalt [28].

#### 2.3.4. BBR

**Figure 9:**
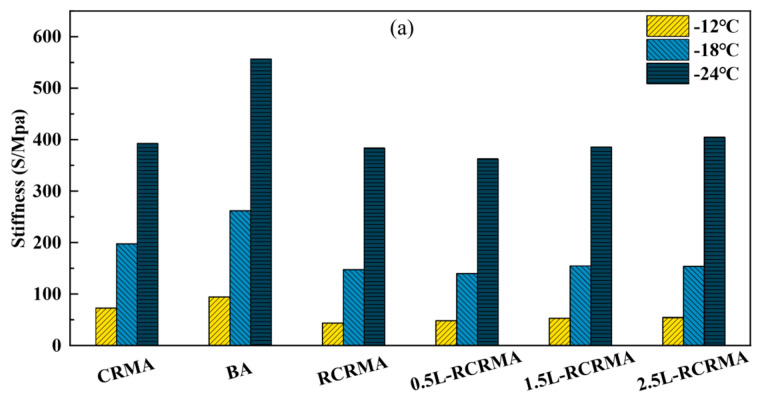

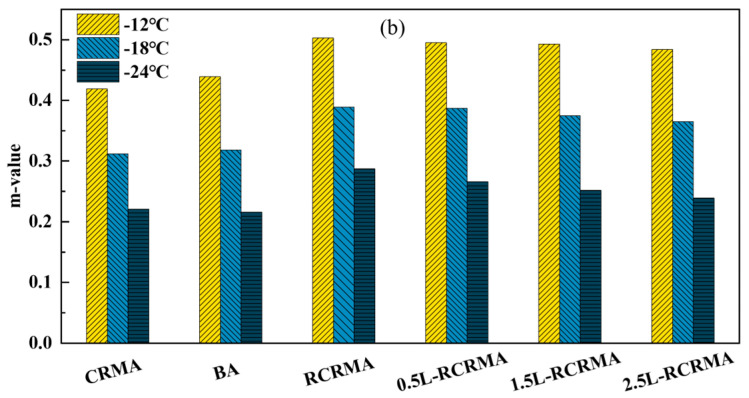
BBR-based assessment of (**a**) bending beam creep modulus of strength (S) and (**b**) bending beam creep rate (m).

We analysed the low-temperature creep of modified asphalt using BBR characterisation. As shown in Figure 9a, the flexural creep modulus (S) of each asphalt sample increased with decreasing temperatures. The LDPE blend generally increased the creep modulus of L-RCRMA and improved the ability of the composite-modified asphalt to resist deformation at low temperatures. The creep modulus of L-RCRMA increased with the increasing LDPE dosage, and 0.5% L-RCRMA had the smallest creep modulus, indicating that this dosage of LDPE can have a beneficial effect on the low-temperature cracking resistance of composite-modified asphalt. The creep moduli of 1.5% L-RCRMA and 2.5% L-RCRMA were slightly higher than that of RCRMA, indicating that the addition of LDPE at higher levels would have a negative effect on the low-temperature cracking resistance of asphalt, but it still had a significant advantage over CRMA and matrix asphalt. As shown in Figure 9b, the flexural creep rate (m) of each asphalt sample decreased with decreasing temperatures. The incorporation of higher dosages of LDPE reduces the flexural creep rate of L-RCRMA and weakens the ability of the asphalt to resist stress dissipation at low temperatures. This is also consistent with the creep modulus results. In summary, the incorporation of LDPE has some negative effects on the low-temperature cracking resistance of composite-modified asphalt, but the effects are mainly manifested at more extreme low temperatures.

## 3. Materials and Methods

### 3.1. Materials

The raw materials required for this study included 60-mesh CR, LDPE, industrial naphthenic oil, matrix asphalt, and DZ catalyst. Sixty-mesh CR was purchased from Dujiangyan Huayi Rubber Co., Ltd. (Chengdu, China) and is produced from scrap tyres by removing impurities and then grinding them; the specific properties are shown in Table 1. LDPE is a white translucent pellet produced in Basel, Switzerland, with its specific properties shown in Table 2. Industrial naphthenic oil was purchased from PetroChina Karamay Petrochemical Company (Karamay, China), and the asphalt belonged to the same petroleum derivatives at room temperature for the brown liquid; the specific properties are shown in Table 3. The asphalt used in the test was 70# A grade matrix asphalt produced by Yunnan Petrochemical (Kunming, China), and all the indices are in accordance with the Technical Specification for Highway Asphalt Pavement Construction JTG F40-2011; the specific properties are shown in Table 4. The DZ catalyst was a mixture of 2,2′-dibenzoylaminodiphenyl disulphide (DBD) and zinc oxide (ZnO), which was derived from the group’s previous research to be effective in assisting desulphurisation at the ratio determined for the test [20].

### 3.2. Sample Preparation

#### 3.2.1. Activation Pre-Treatment of CR and Preparation of LDPE

**Figure 10:**
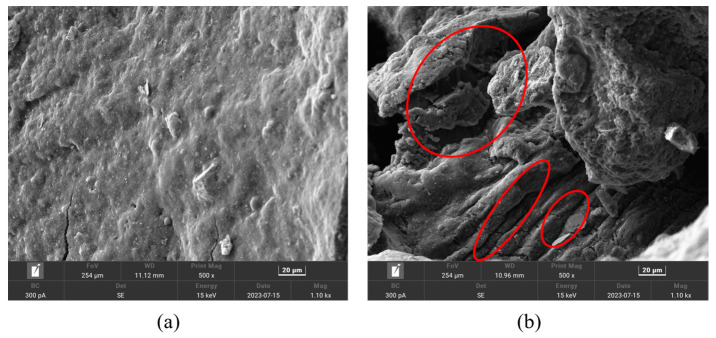
SEM images: (**a**) CR and (**b**) RCR.

During vulcanisation, the carbon atoms of the rubber’s main chain are linked by sulphur bridges to form a cross-linked structure, which provides good elastic properties while preventing the chain from moving independently [29]. As a result, vulcanised rubber is more stable in nature, which makes it difficult to recycle and reuse. In this study, CR was activated and pre-treated by mechanochemical means, disrupting the internally cured cross-linked network structure of CR and reducing it to a linearly structured rubber molecular chain with fluidity and plasticity [30]. Industrial naphthenic oils were used as pre-treatment solvents. Throughout the treatment process, CR was fully heated in the naphthenic oils and underwent solubilisation and degradation reactions. As shown in Figure 10a, the untreated raw CR surface was smooth and flat, which is not easy to use in asphalt binding. As shown by the red label in Figure 10b, the RCR surface after pre-treatment had a rich laminar structure with more holes, which is easier for use in asphalt binding reactions [31,32]. The CR was first weighed and placed in a container. A certain mass of homemade catalyst DZ was weighed on an electronic balance and added to the container. Next, the naphthenic oil was heated in an oven at 100 °C until it became liquid, then proportionally added to the container and mixed with the CR and the catalyst DZ. The container with the sample was placed in the oven for heating and holding; the purpose of this was to heat the sample evenly so that the reaction could take place in the pre-desulphurisation treatment. Finally, the container with heat preservation was placed in the oil bath, and the stirrer was activated; the desulphurisation temperature was 160 °C, the desulphurisation time was 2 h, and the stirrer rotation speed was 450 r/min.

LDPE, as a type of plastic, is difficult to dissolve in asphalt and tends to delaminate when first prepared, so the storage stability of most LDPE-modified asphalts is poor. Before the modified asphalt was prepared, the LDPE was heated and stirred in an oven at 160 °C to soften and homogenise it, and then, it was slowly added to the asphalt, gram-by-gram.

#### 3.2.2. Preparation of LDPE/RCR-Modified Asphalt (L-RCRMA)

**Figure 11:**
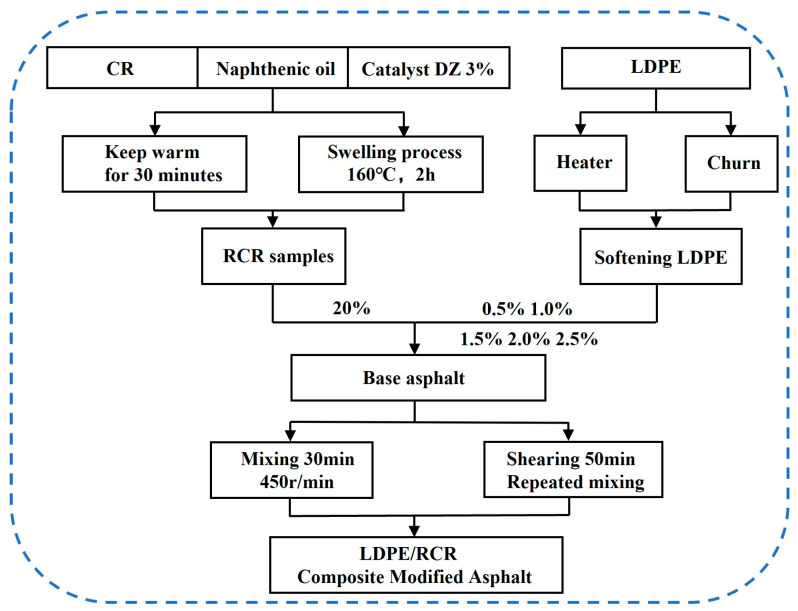
Preparation flow chart.

The preparation process of L-RCRMA is shown in Figure 11. The matrix asphalt was heated to a liquid state, the prepared RCR was slowly added to the asphalt, the mixer was started at 100 rpm, and, after 10 min of mixing, the heated LDPE was added to the asphalt at an even rate and mixed for 30 min. Then, the samples were subjected to shear treatment at a rate of 2500 rpm and a shear time of 50 min, and finally, the prepared samples were developed in an oven for 20 min. The samples were labelled as 0.5L-RCRMA, 1.0L-RCRMA, 1.5L-RCRMA, 2.0L-RCRMA, and 2.5L-RCRMA, according to the amount of LDPE blended into them. The CR was weighed proportionally and poured into the heated asphalt, and the mixer was started at 100 rpm, and after mixing for 10 min, the samples were sheared with a shearer at 2500 rpm for 50 min, and finally, the samples were baked in an oven for 20 min. The samples prepared were CR-modified asphalt, designated CRMA.

### 3.3. Testing and Characterisation

According to the Technical Specification for Highway Asphalt Pavement Construction in China (JTG F40-2011) [33], softening point, 5 °C ductility, 25 °C penetration, and rotational viscosity tests were carried out to determine the properties of the modified asphalt. We placed the sample ring, containing the sample and the sample base plate, in a constant-temperature water bath at 5 °C for at least 15 min, then removed the sample ring and placed it in the instrument to ensure the instrument data were accurate, started the electromagnetic oscillating stirrer, and began heating for the softening point test. We prepared the test samples according to the protocol, fixed the insulated specimens on the metal columns of the elongometer slide and fixing plate, and removed the side moulds. We started the elongometer and observed the extension of the specimen to determine the elongation of the sample. We removed the sample dish to reach a constant temperature, placed the sample dish on the platform of the penetration meter, slowly lowered the needle connecting rod, and used the appropriate position of the reflector or light reflection observation so that the tip of the needle was exactly in contact with the surface of the sample. We reset the displacement meter or dial pointer to zero so as to determine the degree of penetration of the sample needle. The sample was divided into containers for heat preservation. The sample container was removed after levelling the instrument and stirred appropriately; we added the appropriate asphalt sample according to the volume required by the rotor model, held the rotor and sample for 1.5 h, selected the appropriate rotor speed, and began the rotational viscosity test.

The samples were subjected to temperature and frequency scans using a Dynamic Shear Rheometer (DSR) to evaluate their rheological properties over the temperature range. The tests were carried out using 25 mm diameter plates with 1 mm gap, a shear rate of 10 rad/s, and test temperatures ranging from 46 to 82 °C in 6 °C increments. The tests covered a frequency range from 0.1 rad/s to 100 rad/s [34].

Multiple Stress Creep Recovery (MSCR) is an effective method for evaluating the elastic responses of viscoelastic materials [35]. The elastic response of asphalt samples under creep and recovery effects was tested at two stress levels, 0.1 kPa and 3.2 kPa. The test temperature was 58 °C, and the stress was applied for 1 s and then removed, and we waited for a recovery cycle of 9 s. There were 20 cycles, of which the first 10 cycles were used to condition the specimens and the last 10 cycles were used to analyse the data. The average percentage recovery (*R*_0.1_) and unrecoverable creep compliance (*J_nr_*_0.1_) were obtained for all prepared samples during the cycle at 0.1 kPa and at 3.2 kPa. The average percentage recovery (*R*_3.2_) and non-recoverable creep compliance (*J_nr_*_3.2_) were obtained. The calculation is shown in Equations (1)–(6):

(1) $$R\left(\sigma ,N\right)=\frac{\varepsilon_{c}-\varepsilon_{r}}{\varepsilon_{c}-\varepsilon_{0}}\times 100$$

(2) $$J_{nr}\left(\sigma ,N\right)=\frac{\varepsilon_{r}-\varepsilon_{0}}{\sigma }$$

(3) $$R_{0.1}=\frac{\sum_{N=11}^{20}\;R(0.1,N)}{10}$$

(4) $$J_{nr0.1}=\frac{\sum_{N=11}^{20}\;J_{nr}\left(0.1,N\right)}{10}$$

(5) $$R_{3.2}=\frac{\sum_{N=1}^{10}\;R(3.2,N)}{10}$$

(6) $$J_{nr3.2}=\frac{\sum_{N=1}^{10}\;J_{nr}(3.2,N)}{10}$$

where *σ* is the applied stress; *N* is the number of creep and recovery cycles; and *ε* is the shear strain.

The bending beam creep (BBR) test is an effective method for evaluating the low-temperature performance of asphalt [36]. The creep modulus S and creep rate m of asphalt specimens can reflect the low-temperature cracking resistance of asphalt; the lower the creep modulus, the higher the creep rate, indicating that the low-temperature performance of asphalt is better. BBR tests were carried out on matrix asphalt and modified asphalt samples using standard test methods at −12 °C, −18 °C, and −24 °C. S and m were recorded at 60 s.

## 4. Conclusions

In this study, pre-treated CR and LDPE were used for asphalt modification. Based on the three main indicators, rotational viscosity, dynamic shear rheology, and bending beam creep experiments to evaluate the comprehensive physical properties of different dosages of L-RCRMA, the specific conclusions that were reached are as follows:

(1) Rheological experiments showed that the pre-treatment of CR degraded the high-temperature performance of modified asphalt but improved its low-temperature performance and storage stability. On the contrary, with an increase in LDPE doping, the high-temperature rheological properties of L-RCRMA showed an increasing trend and the low-temperature rheological properties showed a decreasing trend, but the low-temperature rheological properties decreased less significantly.
(2) The incorporation of RCR and LDPE improved the rotational viscosity of L-RCRMA at all temperatures, which was significantly better than that of CRMA.
(3) The Han curve showed that in the range of 0.5% to 2.5%, the incorporation of LDPE had no negative effect on the original good compatibility of RCRMA, and the Han curve of L-RCRMA remained smooth and continuous.
(4) Compared with RCRMA, 1.5% L-RCRMA has obvious high-temperature stability and maintained good compatibility; compared with CRMA, it has obvious low-temperature crack resistance, is environmentally friendly, and has excellent performance as a new road material. In summary, we suggest that the ideal dosage of RCR is 20%, and the ideal dosage of LDPE is 1.5%.

## Figures and Tables

**Table 1:** Basic properties of CR.

Parameters	Heating Reduction /%	Ash Content /%	Iron Content /%	Fibre Content /%	Sieve Residue /%	Bulk Density /kg/m^3^
Test Value	0.62	8.75	0.029	0	0.014	314
Experimental Methods	GB/T19208-2008	GB/T4498-2013	GB/T19208-2008	GB/T19208-2008	GB/T19208-2008	GB/T19208-2008

**Table 2:** Basic properties of LDPE.

Parameters	Density /g·cm^−3^	Melting Point /°C	Tensile Strength /MPa
Test Value	0.936	123	9.2
Experimental Methods	GB/T2559-2005	GB/T8026-2014	ASTM D882

**Table 3:** Basic properties of naphthenic oils.

Parameters	Density /g·cm^−3^	Viscosity /m^2^·s^−1^ (100 °C)	Saturated Phenol /%
Test Value	1.02	40	87
Experimental Methods	GB/T1884-92	GB/T265-88	ASM-IP-2

**Table 4:** Basic properties of asphalt.

Parameters	Softening Point /°C	Ductility (15 °C)/cm	Penetration (25 °C)/mm	Flash Point /°C
Test Value	45.8	>100	70.5	286
Standard Value	≥45	≥100	60~80	≥260
Experimental Methods	GB/T0606-2011	GB/T0605-2011	GB/T0604-2011	GB/T0611-2011

## Data Availability

The data presented in this study are available from the corresponding author upon reasonable request.
